# Artificial Intelligence in Lymphoma Histopathology: Systematic Review

**DOI:** 10.2196/62851

**Published:** 2025-02-14

**Authors:** Yao Fu, Zongyao Huang, Xudong Deng, Linna Xu, Yang Liu, Mingxing Zhang, Jinyi Liu, Bin Huang

**Affiliations:** 1 Sichuan Tianfu New Area People's Hospital Chengdu China; 2 Department of Pathology Sichuan Clinical Research Center for Cancer, Sichuan Cancer Hospital & Institute Sichuan Cancer Center, University of Electronic Science and Technology of China Chengdu China; 3 Wonders Information Co., Ltd Shanghai China; 4 Phase I Clinical Trial Unit Sichuan Clinical Research Center for Cancer, Sichuan Cancer Hospital & Institute Sichuan Cancer Center, University of Electronic Science and Technology of China Chengdu China

**Keywords:** lymphoma, artificial intelligence, bias, histopathology, tumor, hematological, lymphatic disease, public health, pathologists, pathology, immunohistochemistry, diagnosis, prognosis

## Abstract

**Background:**

Artificial intelligence (AI) shows considerable promise in the areas of lymphoma diagnosis, prognosis, and gene prediction. However, a comprehensive assessment of potential biases and the clinical utility of AI models is still needed.

**Objective:**

Our goal was to evaluate the biases of published studies using AI models for lymphoma histopathology and assess the clinical utility of comprehensive AI models for diagnosis or prognosis.

**Methods:**

This study adhered to the Systematic Review Reporting Standards. A comprehensive literature search was conducted across PubMed, Cochrane Library, and Web of Science from their inception until August 30, 2024. The search criteria included the use of AI for prognosis involving human lymphoma tissue pathology images, diagnosis, gene mutation prediction, etc. The risk of bias was evaluated using the Prediction Model Risk of Bias Assessment Tool (PROBAST). Information for each AI model was systematically tabulated, and summary statistics were reported. The study is registered with PROSPERO (CRD42024537394) and follows the PRISMA (Preferred Reporting Items for Systematic Reviews and Meta-Analyses) 2020 reporting guidelines.

**Results:**

The search identified 3565 records, with 41 articles ultimately meeting the inclusion criteria. A total of 41 AI models were included in the analysis, comprising 17 diagnostic models, 10 prognostic models, 2 models for detecting ectopic gene expression, and 12 additional models related to diagnosis. All studies exhibited a high or unclear risk of bias, primarily due to limited analysis and incomplete reporting of participant recruitment. Most high-risk models (10/41) predominantly assigned high-risk classifications to participants. Almost all the articles presented an unclear risk of bias in at least one domain, with the most frequent being participant selection (16/41) and statistical analysis (37/41). The primary reasons for this were insufficient analysis of participant recruitment and a lack of interpretability in outcome analyses. In the diagnostic models, the most frequently studied lymphoma subtypes were diffuse large B-cell lymphoma, follicular lymphoma, chronic lymphocytic leukemia, and mantle cell lymphoma, while in the prognostic models, the most common subtypes were diffuse large B-cell lymphoma, follicular lymphoma, chronic lymphocytic leukemia, and Hodgkin lymphoma. In the internal validation results of all models, the area under the receiver operating characteristic curve (AUC) ranged from 0.75 to 0.99 and accuracy ranged from 68.3% to 100%. In models with external validation results, the AUC ranged from 0.93 to 0.99.

**Conclusions:**

From a methodological perspective, all models exhibited biases. The enhancement of the accuracy of AI models and the acceleration of their clinical translation hinge on several critical aspects. These include the comprehensive reporting of data sources, the diversity of datasets, the study design, the transparency and interpretability of AI models, the use of cross-validation and external validation, and adherence to regulatory guidance and standardized processes in the field of medical AI.

## Introduction

Lymphoma, a malignancy that originates from the lymphohematopoietic system, is recognized as one of the most prevalent hematological cancers globally. Epidemiological studies indicate that Hodgkin lymphoma (HL) and non-Hodgkin lymphoma (NHL) are prevalent malignant lymphatic disorders that pose significant public health challenges. Data from GLOBOCAN 2020 reveal that the projected global incidence and mortality for HL are 83,087 and 23,376 cases, respectively, while for NHL, these figures stand at 544,352 and 259,793, respectively [1,2]. In China, lymphoma is a considerable public health challenge. Data from the Global Burden of Diseases (GBD), Injuries, and Risk Factors Study for 2019 reveal that the age-standardized incidence rate for HL is 0.57 cases per 100,000 individuals, with an age-standardized mortality rate of 0.15 per 100,000 individuals. For NHL, the age-standardized incidence rate is significantly higher at 4.99 per 100,000 individuals, and the age-standardized mortality rate is 2.32 per 100,000 individuals [3].

Histopathology, which involves examining tissue specimens at the cellular level, is the gold standard for the diagnosis of lymphomas [4]. The conventional diagnostic process typically involves pathologists using hematoxylin-eosin (HE) staining of tissues and immunophenotyping for diagnosis. For the diagnosis of high-grade B-cell lymphomas, fluorescence in situ hybridization (FISH) is also commonly employed in conjunction [5]. However, this method has several drawbacks, including subjectivity, time-consuming procedures, and high costs [6].

Traditionally, pathologists have relied on optical microscopes to analyze pathological tissue sections. However, the advent of digital pathology has seen a shift toward the use of computers for reviewing and analyzing scanned whole slide images (WSIs). This transition is not only driven by the potential for increased efficiency but also opens new avenues for the development of automated diagnostic tools [7]. These tools have the potential to enhance the accuracy, efficiency, objectivity, and consistency of diagnoses, which is crucial in addressing the global shortage of pathologists. They can also increase diagnostic throughput and reduce the reliance on referrals and additional tests [8]. This field of research is burgeoning, and for certain types of malignant tumors, these systems are beginning to demonstrate clinical utility [9]. However, although many research models of artificial intelligence (AI) applied to lymphoma histopathology have been published, it is unclear whether there are methodological biases in these models, and the clinical utility of AI applied to lymphoma histopathology has not been summarized.

In this extensive study, we systematically reviewed the literature exploring the use of AI technologies, including traditional machine learning (ML) and deep learning (DL) methods, to assess digital pathology images for lymphoma diagnosis, prognosis, and other pertinent applications. Our review encompasses research that focuses on individual diagnostic factors, such as histological subtypes, as well as studies that perform computer-assisted tasks like tumor segmentation. We also assessed the clinical utility of these AI methods with consideration of potential biases. The objective of this review is to provide valuable insights and actionable recommendations based on the existing body of literature. Thus, this review aims to provide insights and recommendations based on published literature to improve the clinical utility of future research, including reducing the risk of bias, improving reproducibility, and increasing generalizability.

## Methods

### Literature Search

A thorough search was conducted across 3 prominent research databases: PubMed, Cochrane, and Web of Science. The search was restricted to peer-reviewed journals and conference proceedings to ensure the quality and credibility of the studies included. The search timeline extended from the inception of each database up to August 30, 2024. We employed MeSH terms for more precise retrieval.

Given the multitude of terms related to AI, our search strategy incorporated keywords such as “artificial intelligence,” “machine learning,” “neural network,” and “network, neural (computer),” along with “lymphoma.” We combined multiple relevant terms for each concept using the OR operator (eg, “artificial intelligence” OR “machine learning”) and then merged “lymphoma” with “artificial intelligence” using the AND operator. This approach ensured that the retrieved studies met both criteria.

Subsequently, we screened the articles based on their relevance to histopathological AI, focusing on the title and abstract. The review protocol was registered on PROSPERO (CRD42024537394) prior to the screening of search results for inclusion. Detailed search strategies and methods are provided in Multimedia Appendix 1.

### Literature Selection

A researcher (YF) manually removed duplicate papers with the assistance of the reference management software EndNote 20. Subsequently, another researcher (ZH) independently screened the articles for inclusion in 2 stages: the first based on titles and abstracts, and the second based on the full text. Disagreements were discussed and arbitrated by a third researcher (JL).

The inclusion criteria required the research to evaluate the use of at least one AI approach to make diagnostic or prognostic inferences on human histopathology images from suspected or confirmed cases of lymphoma. Studies were only included if AI methods were applied directly to the digital pathology images or to features that were automatically extracted from the images. Fundamental tasks, such as segmentation and cell counting, were included as these could be used by pathologists for computer-aided diagnosis. Only conventional light microscopy images were considered, with other imaging modalities, such as fluorescence and hyperspectral imaging, excluded. Publications that did not include primary research, such as review papers, were excluded. Non-English language articles and research where a full version of the manuscript was not accessible were excluded.

In the studies included, models were deemed of interest if they adhered to the same inclusion criteria. When several models were compared against the same outcome, the model of interest was typically the newly proposed one. If this was ambiguous, the model with the best performance during the validation phase was selected. When multiple models from a single study exhibited similar modeling techniques, the one with superior validation performance was included in the assessment. Results from the same model at varying levels of precision (eg, patch level, slice level, and patient level) were not treated as distinct outcomes.

### Risk of Bias Assessment

The risk of bias in the models of interest was assessed using the Prediction Model Risk of Bias Assessment Tool (PROBAST) [10]. The tool evaluates the likelihood that the reported results are distorted due to limitations in study design, conduct, and analysis. PROBAST includes 20 guiding questions categorized into 4 domains: Participants, Predictors, Outcomes, and Analysis. These questions are summarized to indicate a high risk or low risk of bias or are marked as unclear when insufficient information is available for a comprehensive assessment and no information is available to suggest a high risk of bias. It is important to note that an unclear risk of bias does not imply a methodological flaw but rather indicates incomplete reporting.

The Participants domain involves the recruitment and selection of participants to ensure the consistency and representativeness of the study population targeting the intended demographic. Relevant details include the recruitment strategy, inclusion criteria, and number of participants enrolled.

The Predictors domain addresses the consistent definition and measurement of predictive variables, which in this context often refers to the generation of digital pathology images. This encompasses methods for the fixation, staining, scanning, and digital processing of tissues prior to modeling.

The Outcomes domain involves the appropriate definition and consistent determination of ground truth labels. This includes the criteria used to ascertain diagnoses or prognoses, the expertise of those determining these labels, and whether the labels are independent of any model outputs.

The Analysis domain encompasses statistical considerations in the evaluation of model performance to ensure valid and not overly optimistic results. It includes various factors, such as the number of participants for each outcome in the test set, the validation methods used (cross-validation, external validation, internal validation, etc), the metrics for assessing performance, and the methods to address the impact of censoring, confounding, and missing data. Some of these factors are interrelated. For example, the risk of bias due to a small dataset is somewhat mitigated by cross-validation, which increases the effective size of the test set and can be used to assess variability, reducing the optimism of the results. Additionally, the risk associated with using a small dataset depends on the type of outcome being predicted; robust analysis for a 5-class classification requires more data than a binary classification. There must also be sufficient data across all relevant patient subgroups; for instance, if multiple subtypes of lymphomas are included, it is not acceptable for 1 subtype to be represented by only a few patients. Due to these interrelated factors, there are no rigid standards for determining the appropriate size of a dataset.

Inconsistencies in methodology often lead to bias risk. For example, inconsistencies in HE staining from different research centers can lead to heterogeneity in the visual characteristics of digital pathology slides, potentially causing spurious correlations through random or systematic differences within or between subgroups in the dataset. Using a large dataset during training may enhance the model’s generalizability, but this must be tightly controlled to avoid introducing systematic confounding. Inconsistencies in the determination of outcomes may mean that the results of a study are unreliable due to spurious correlations in the underlying factual labels or invalid due to misjudgment of the labels.

While PROBAST provides a framework for assessing the risk of bias, there is a degree of subjectivity in interpreting the signal questions. Therefore, each model was analyzed by 2 independent researchers (YF and ZH), with at least one computer scientist and one pathologist involved in the bias risk assessment of each model.

The Quality Assessment of Diagnostic Accuracy Studies-AI (QUADAS-AI) tool was used to evaluate the sensitivity of the included studies. QUADAS-AI is the AI-specific extension of QUADAS-2 [11] and QUADAS-C [12], and includes 4 domains for determining the risk of bias (patient selection, reference standard, index test, and flow and timing) and 3 domains for applicability issues (index test, patient selection, and reference standard) (Multimedia Appendix 2).

### Data Synthesis

Data extraction was independently performed by 2 researchers (YF and MZ), using a form containing 67 fields within the categories Overview, Data, Methods, Results, and Miscellaneous. A summary of this process is provided in Multimedia Appendix 3.

Information was sought from full-text articles, as well as references and supplementary materials where appropriate. Inferences were made only when both researchers were confident that this gave the correct information, with disagreements resolved through discussion. Fields that could not be confidently completed were labeled as being unclear.

All extracted data were summarized in 2 tables, 1 each for study-level and model-level characteristics. Only models of interest were included in these tables. The term model outcome refers to the model output (whether this was a clinical outcome [diagnosis or prognosis] or a diagnostically relevant outcome that could be used for computer-aided diagnosis, such as tumor segmentation). The data synthesis did not include any meta-analysis due to the diversity of the included methods and model outcomes. The PRISMA (Preferred Reporting Items for Systematic Reviews and Meta-Analyses) 2020 guidelines for reporting systematic reviews were followed, with checklists provided in Multimedia Appendix 3.

## Results

### Results of Literature Screening

**Figure 1:**
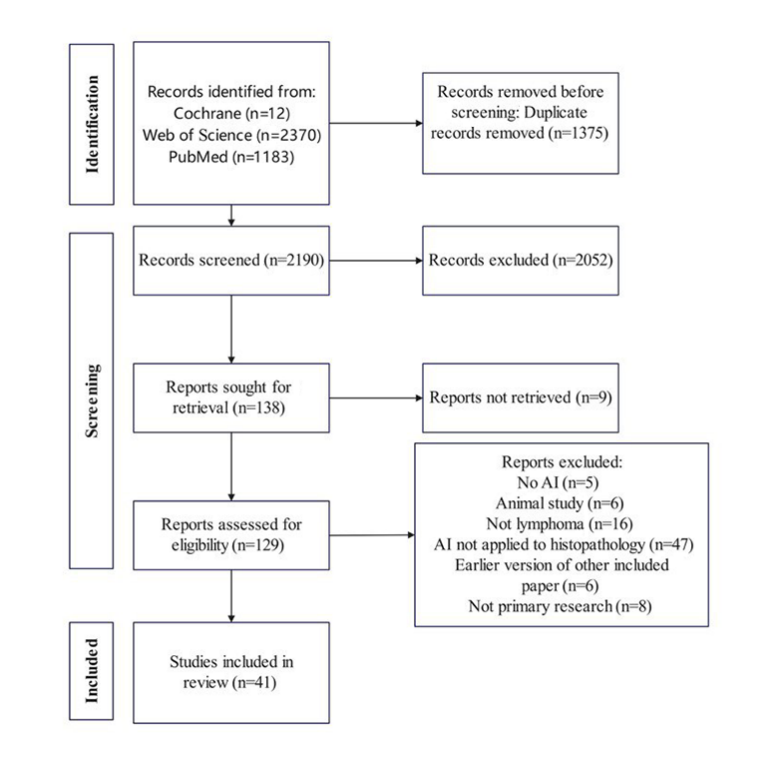
PRISMA (Preferred Reporting Items for Systematic Reviews and Meta-Analyses) 2020 flowchart. AI: artificial intelligence.

As shown in Figure 1, the initial literature search identified a total of 3565 records, with 1375 being duplicates. After screening titles and abstracts, 2052 records were excluded, leaving 41 studies for inclusion in the review. All studies that met the inclusion criteria were identified through research databases, with no eligible records found in trial registries. Although the search was performed from 1949, all included studies were published since 2010, and more than 80% were published since 2020. The characteristics of these studies are summarized in Table 1. The model construction parameters of the 41 included studies are detailed in Table 2.

**Table 1:** Characteristics of the 41 studies included in this systematic review.

Publication	Data source	Geographical distribution	Internal participants, n	Internal pathology images, n	Subtypes	Outcome type	Model task	Code available
Achi et al [13], 2019	Single center	America	128	2560	Burkitt lymphoma, DLBCL^a^, and small lymphocytic lymphoma	Diagnosis	Identification	None
Steinbuss et al [14], 2021	Single center	Germany	629	Unclear	Nodal small lymphocytic lymphoma/CLL^b^ and DLBCL	Diagnosis	Classification	[15]
Miyoshi et al [16], 2020	Single center	Japan	388	Unclear	FL^c^ and DLBCL	Diagnosis	Classification	None
Syrykh et al [17], 2020	Single center	France	378	Unclear	FL	Diagnosis	Identification	[18]
Sereda et al [19], 2023	Single center	Germany	53	Unclear	Pediatric nodular lymphocyte–predominant Hodgkin lymphoma	Prognosis	Identification, semantic segmentation	None
Zhang et al [20], 2020	Single center	China	Unclear	374	CLL, MCL^d^, and FL	Diagnosis	Classification	None
Li et al [21], 2022	Multicenter	America	1005	3123	DLBCL	Diagnosis	Classification	[22]
Zhang et al [23], 2021	Single center	China	Unclear	374	CLL, MCL, and FL	Other	Classification	None
Yu et al [24], 2021	Multicenter	China	40	40	Monomorphic epitheliotropic intestinal T-cell lymphoma	Other	Classification, cell nuclei segmentation	None
Takagi et al [25], 2023	Single center	Japan	842	Unclear	DLBCL and FL	Other	Classification	None
Hamdi et al [26], 2023	Single center	Saudi Arabia	Unclear	15,000	CLL, MCL, and FL	Diagnosis	Classification	[27]
Mohlman et al [28], 2020	Single center	America	70	10,818	DLBCL and Burkitt lymphoma	Other	Classification	None
Karabulut et al [29], 2023	Single center	Turkey	11	60	MF^e^	Diagnosis	Identification, classification, cell nuclei segmentation	None
do Nascimento et al [30], 2018	Single center	Brazil	347	15,353	CLL, MCL, and FL	Diagnosis	Classification	None
Zhu et al [31], 2019	Multicenter	China	Unclear	374	CLL, MCL, and FL	Other	Classification	None
Hashimoto et al [32], 2022	Single center	Japan	262	Unclear	DLBCL, Burkitt lymphoma, and FL	Other	Classification	None
Michail et al [33], 2014	Single center	Greece	Unclear	300	FL	Other	Identification, semantic segmentation	None
Kornaropoulos et al [34], 2014	Single center	Greece	17	500	FL	Other	Classification	None
Chuang et al [35], 2022	Single center	China	103	309	MCL	Prognosis	Semantic segmentation	None
Vrabac et al [36], 2021	Multicenter	America	209	Unclear	DLBCL	Prognosis	Semantic segmentation	[37]
Motmaen et al [38], 2023	Single center	Germany	83	Unclear	HL^f^	Prognosis	Classification	None
Swiderska-Chadaj et al [39], 2021	Multicenter	Holland	287	354	DLBCL	MYC translocation detection	Identification	None
Tagami et al [40], 2023	Single center	Japan	129	1290	Ocular adnexal mucosa–associated lymphoid tissue lymphoma	Other	Classification	None
Irshaid et al [41], 2022	Single center	America	61	Unclear	CLL and FL	Prognosis	Cell nuclei segmentation, classification	None
El Hussein et al [42], 2022	Multicenter	America	125	213	CLL	Prognosis	Cell nuclei segmentation, classification	None
Chen et al [43], 2022	Multicenter	America	135	213	CLL	Prognosis	Cell nuclei segmentation, classification	None
Zhang et al [44], 2024	Multicenter	China	Unclear	Unclear	Primary central nervous system lymphoma	Diagnosis	Classification	[45]
Tavolara et al [46], 2024	Single center	America	172	376	DLBCL	Other	Identification	[47]
Perry et al [5], 2023	Single center	Israel	57	Unclear	High-grade B-cell lymphoma	Diagnosis	Classification	None
Yan et al [48], 2024	Single center	China	220	220	DLBCL	Prognosis	Cell nuclei segmentation	[49]
Lee et al [50], 2024	Single center	America	216	251	DLBCL	Prognosis	Cell nuclei segmentation	None
Duan et al [51], 2024	Single center	China	114	132	Primary central nervous system lymphoma	Prognosis	Cell nuclei segmentation	None
Quan et al [52], 2024	Single center	China	Unclear	350	Gastric MALT^g^ lymphoma	Diagnosis	Cell nuclei segmentation	None
Al-Mekhlafi et al [53], 2022	Single center	Saudi Arabia	Unclear	1500	CLL, FL, and MCL	Diagnosis	Classification	None
Somaratne et al [54], 2019	Single center	Australia	Unclear	374	FL	Diagnosis	Classification	None
Codella et al [55], 2016	Multicenter	America	Unclear	374	CLL, FL, and MCL	Diagnosis	Classification	None
Swiderska-Chadaj et al [56], 2020	Multicenter	America	91	157	DLBCL	MYC translocation detection	Identification	None
Basu et al [57], 2022	Unclear	India	Unclear	700	MF	Diagnosis	Classification	None
Shankar et al [58], 2023	Single center	America	Unclear	Unclear	cHL, DLBCL, and MCL	Diagnosis	Classification	[59]
Soltane et al [60], 2022	Unclear	Saudi Arabia	Unclear	323	cHL, nodular lymphoma predominant, Burkitt lymphoma, FL, MCL, large B-cell lymphoma, and T-cell lymphoma	Diagnosis	Classification	None
Tagami et al [61], 2024	Single center	Japan	127	1270	Orbital MALT lymphoma	Diagnosis	Classification	None

^a^DLBCL: diffuse large B-cell lymphoma.

^b^CLL: chronic lymphocytic leukemia.

^c^FL: follicular lymphoma.

^d^MCL: mantle cell lymphoma.

^e^MF: mycosis fungoides.

^f^HL: Hodgkin lymphoma.

^g^MALT: mucosa-associated lymphoid tissue.

**Table 2:** Model construction parameters for the 41 included studies.

Publication	Stain type	Original image size	Patch size	Magnification	Feature extraction	Final model	Final model prediction precision	Validation type	External validation data	Metric	Internal results	External results
Achi et al [13]	HE^a^	WSI^b^	40×40	40×	Learned	CNN^c^	Patch	Internal validation	None	Accuracy	95%	None
Steinbuss et al [14]	HE	WSI	395×395	40×	Hand-crafted	Efficient Net	Patch	Internal validation	None	Accuracy	95%	None
Miyoshi et al [16]	HE	WSI	64×64	5×	Hand-crafted	CNN	Patch	5-fold cross-validation	None	Accuracy	94%	None
Syrykh et al [17]	HE	WSI	299×299	20×	Hand-crafted	BNN^d^	Patch	Internal validation	None	AUC^e^	0.92-0.99	None
Zhang et al [20]	HE	WSI	224×224	Unclear	Learned	ResNet-50	Patch	5-fold cross-validation	None	Accuracy	95.4%	None
Li et al [21]	HE	WSI	945×945	40×	Learned	17CNN+transform	Patch	External validation	402	Accuracy	100%	100%
Hamdi et al [26]	HE	WSI	Unclear	Unclear	Hand-crafted	MobileNet-VGG-16, decision tree–based machine learning	WSI	Internal validation	None	AUC, accuracy	0.99, 99.8%	None
Karabulut et al [29]	HE	Microscopic images	600×600	200×	Learned	DL^f^	Patch	Internal validation	None	Accuracy	94.2%	None
do Nascimento et al [30]	HE	Microscopic images	Unclear	1000×	Unclear	Classification using the polynomial	Unclear	Internal validation	None	Accuracy	96%-100%	None
Sereda et al [19]	IHC^g^	Patch	256×256	Unclear	Hand-crafted	YOLOv4-tiny CNN	Patch	Internal validation	None	Accuracy	95.43%	None
Chuang et al [35]	HE	WSI	132×132	40×	Hand-crafted	CNN	Patch	Internal validation	None	AUC	0.94	None
Vrabac et al [36]	HE	WSI	224×224	40×	Hand-crafted	Hover-Net	Patch	External validation	179	CI	None	95%
Motmaen et al [38]	Picrosirius Red	WSI	320×320	20×	Hand-crafted	YOLOv4	Patch	Internal validation	None	AUC	0.79	None
Irshaid et al [41]	HE	WSI	128×128	40×	Hand-crafted	CNN	Patch	Internal validation	None	AUC	0.85	None
El Hussein et al [42]	HE	WSI	256×256	20×	Hand-crafted	Hover-Net	WSI	External validation	28	AUC	None	0.93
Chen et al [43]	HE	WSI	256×256	20×	Hand-crafted	Hover-Net	WSI	External validation	68	Accuracy	None	92.5%
Swiderska-Chadaj et al [39]	HE	WSI	Unclear	20×	Learned	U-Net	WSI	External validation	49	Sensitivity	None	93%
Zhang et al [23]	HE	WSI	Unclear	Unclear	Hand-crafted	ResNet-50	Patch	Internal validation	None	Accuracy	98.63%	None
Yu et al [24]	HE	WSI	115×115	40×	Hand-crafted	Decision tree–based machine learning	Patch	Internal validation	None	AUC	0.96	None
Takagi et al [25]	HE	WSI	224×224	40×	Learned	CNN	Patch	5-fold cross-validation	None	Accuracy	0.83	None
Mohlman et al [28]	HE	WSI	224×224	200×	Learned	CNN	Patch	Internal validation	None	AUC	0.92	None
Zhu et al [31]	HE	WSI	64×64	Unclear	Learned	VGG-16, LSTM^h^	Patch	10-fold cross-validation	None	Overall grading accuracy	0.98	None
Hashimoto et al [32]	HE	WSI	224×224	20×	Learned	CNN	Patch	5-fold cross-validation	None	Accuracy	68.3%	None
Michail et al [33]	HE	Microscopic images	Unclear	40×	Hand-crafted	SVM^i^	Microscopic images	Internal validation	None	Accuracy	97.4%	None
Kornaropoulos et al [34]	HE	WSI	71×71	Unclear	Hand-crafted	Laplacian Eigenmaps	WSI	Hold-out K-folds	None	Accuracy	99.22%	None
Tagami et al [40]	HE	WSI	2048×2048	20×	Hand-crafted	SVM	Patch	10-fold cross-validation	None	AUC	0.86	None
Zhang et al [44]	HE	WSI	256×256	40×	Learned	DL	Patch	External validation	None	AUC	0.96	None
Tavolara et al [46]	IHC	WSI	224×224	40×	Hand-crafted	ResNet-50	Patch	External validation	108	Sensitivity, specificity	None	0.857, 0.991
Perry et al [5]	HE	WSI	384×384	40×	Hand-crafted	DL	Patch	Internal validation	None	AUC	0.95	None
Yan et al [48]	IHC	WSI	256×256	40×	Hand-crafted	CNN	Patch	External validation	61	ICC^j^	None	96%
Lee et al [50]	HE	WSI	224×224	40×	Learned	ViT-S/8	Patch	External validation	48	Sensitivity, specificity	None	90.2%, 70.0%
Duan et al [51]	HE	WSI	512×512	20×	Hand-crafted	KNN^k^	Patch	Internal validation	46	AUC	None	0.92
Quan et al [52]	HE	WSI	512×512	40×	Hand-crafted	ResNet 50	Patch	5-fold cross-validation	None	Sensitivity, specificity	96.79%, SD 1.50%; 99.38%, SD 0.15%	None
Al-Mekhlafi et al [53]	HE	WSI	512×512	40×	Hand-crafted	ResNet 50	Patch	Internal validation	None	AUC	0.99	None
Somaratne et al [54]	HE	WSI	227×227	Unclear	Hand-crafted	AlexNet	Patch	External validation	213	AUC	None	0.99
Codella et al [55]	HE	WSI	Unclear	Unclear	Unclear	Unclear	Patch	3-fold cross-validation	None	Accuracy	92.3%	None
Swiderska-Chadaj et al [56]	HE	WSI	512×512	20×	Hand-crafted	CNN	Patch	External validation	66	AUC	None	0.83
Basu et al [57]	HE	WSI	224×224	40×	Hand-crafted	CNN	Patch	Internal validation	None	Sensitivity, specificity	94.67%, 97.3%	None
Shankar et al [58]	HE	WSI	Unclear	40×	Hand-crafted	ResNet 50	Patch	Internal validation	None	AUC	0.95	None
Soltane et al [60]	HE	WSI	224×224	Unclear	Learned	ResNet 50	Patch	5-fold cross-validation	None	Accuracy	91.6%	None
Tagami et al [61]	HE	WSI	2048×2048	20×	Hand-crafted	DL	Patch	5-fold cross-validation	None	AUC	0.8	None

^a^HE: hematoxylin-eosin.

^b^WSI: whole slide image.

^c^CNN: convolutional neural network.

^d^BNN: Bayesian neural network.

^e^AUC: area under the receiver operating characteristic curve.

^f^DL: deep learning.

^g^IHC: immunohistochemical.

^h^LSTM: long short-term memory.

^i^SVM: support vector machine.

^j^ICC: intraclass correlation coefficient.

^k^KNN: k-nearest neighbors.

### Risk of Bias Assessment

**Figure 2:**
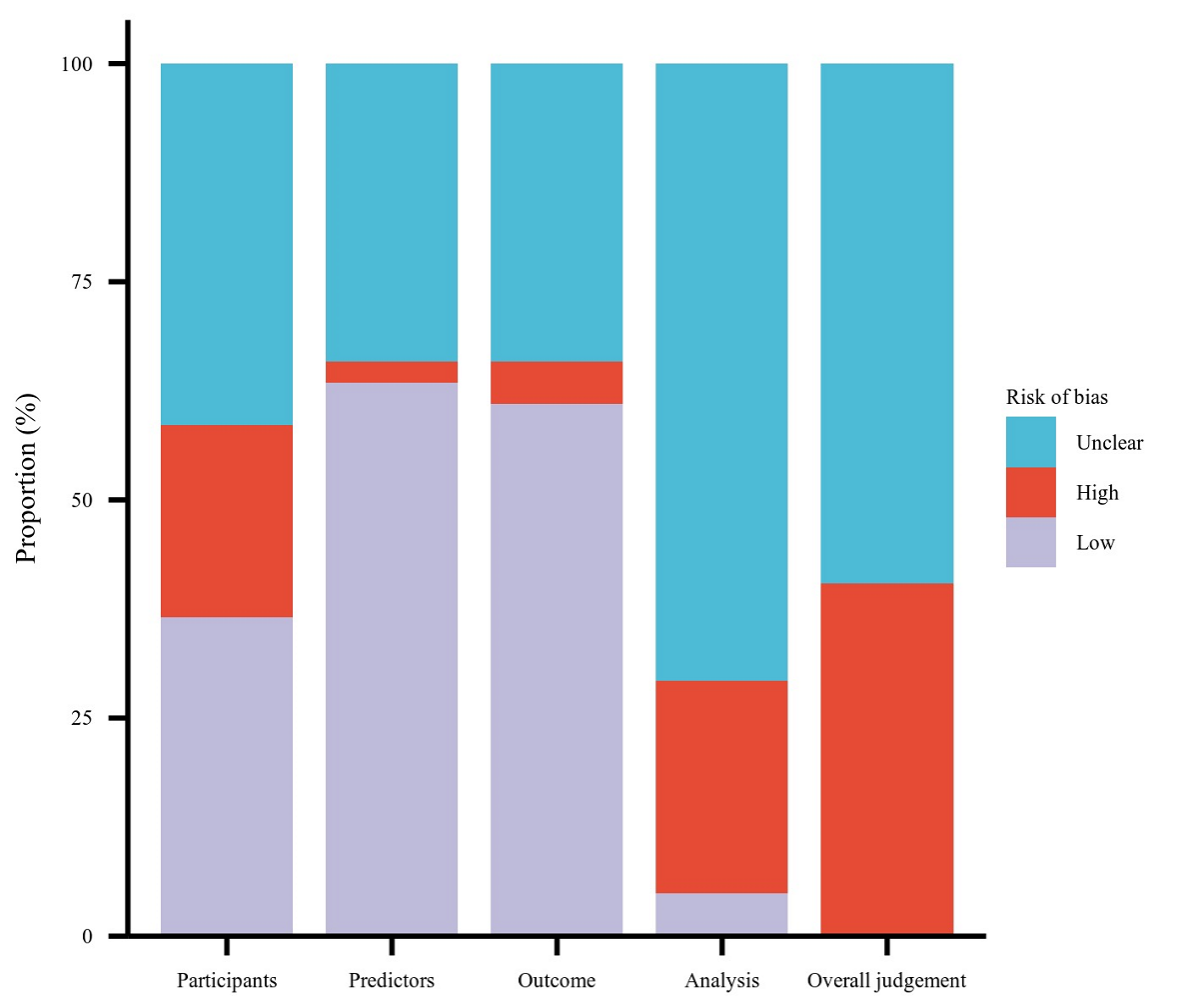
Prediction Model Risk of Bias Assessment Tool (PROBAST) risk of bias.

The PROBAST assessment findings are detailed in Table 3. Despite some studies encompassing multiple models of interest, each paper highlighted 1 model with superior predictive value for bias risk analysis. Most models exhibited either a high overall bias risk (13/41) or an unclear overall bias risk (28/41), with none of the models presenting a low overall bias risk (0/41). Most high-risk models predominantly allocated their high-risk scores in the Participants domain (10/41). Conversely, most low-risk scores were concentrated in the Predictors (26/41) and Outcomes (26/41) domains. Almost all studies reported an unclear risk of bias in at least one domain, with the Participants (16/41) and Statistical Analysis (37/41) domains being the most frequently affected. Qualitative summaries are presented in Figure 2.

**Table 3:** Prediction Model Risk of Bias Assessment Tool (PROBAST) risk of bias assessment results for the 41 papers included in this review.

Publication	Participants	Predictors	Outcomes	Analysis	Overall judgement
Achi et al [13]	Unclear	Low	Unclear	Unclear	Unclear concerns
Steinbuss et al [14]	High	Unclear	Low	Unclear	High concerns
Miyoshi et al [16]	High	Low	Low	Unclear	High concerns
Syrykh et al [17]	High	Low	Low	Low	High concerns
Sereda et al [19]	Low	Low	Low	Unclear	Unclear concerns
Zhang et al [20]	Unclear	Low	Unclear	Unclear	Unclear concerns
Li et al [21]	High	Low	Low	Unclear	High concerns
Zhang et al [23]	Unclear	Unclear	Low	Unclear	Unclear concerns
Yu et al [24]	Unclear	Low	Low	Unclear	Unclear concerns
Takagi et al [25]	Unclear	Low	Low	Unclear	Unclear concerns
Hamdi et al [26]	Unclear	Low	Unclear	Unclear	Unclear concerns
Mohlman et al [28]	High	Low	Low	Low	High concerns
Karabulut et al [29]	Unclear	Low	Low	Unclear	Unclear concerns
do Nascimento et al [30]	Unclear	Unclear	Unclear	Unclear	Unclear concerns
Zhu et al [31]	Unclear	Low	Unclear	Unclear	Unclear concerns
Hashimoto et al [32]	Unclear	Low	Unclear	Unclear	Unclear concerns
Michail et al [33]	Unclear	Unclear	Unclear	Unclear	Unclear concerns
Kornaropoulos et al [34]	Low	Low	Low	Unclear	Unclear concerns
Chuang et al [35]	Unclear	Low	Unclear	Unclear	Unclear concerns
Vrabac et al [36]	Low	Low	Low	Unclear	Unclear concerns
Motmaen et al [38]	High	Unclear	Low	Unclear	High concerns
Swiderska-Chadaj et al [39]	Unclear	Unclear	Unclear	Unclear	Unclear concerns
Tagami et al [40]	High	Unclear	Low	Unclear	High concerns
Irshaid et al [41]	High	Low	Low	Unclear	High concerns
El Hussein et al [42]	High	Low	Low	Unclear	High concerns
Chen et al [43]	High	Low	Low	Unclear	High concerns
Zhang et al [44]	Unclear	Unclear	Low	Unclear	Unclear concerns
Tavolara et al [46]	Low	Low	Unclear	Unclear	Unclear concerns
Perry et al [5]	Low	High	Low	High	High concerns
Yan et al [48]	Low	Low	Low	Unclear	Unclear concerns
Lee et al [50]	Low	Low	Low	Unclear	Unclear concerns
Duan et al [51]	Low	Low	High	High	High concerns
Quan et al [52]	Low	Unclear	Unclear	Unclear	High concerns
Al-Mekhlafi et al [53]	Low	Unclear	Low	Unclear	High concerns
Somaratne et al [54]	Low	Unclear	Low	Unclear	Unclear concerns
Codella et al [55]	Low	Unclear	Unclear	High	High concerns
Swiderska-Chadaj et al [56]	Low	Unclear	Low	Unclear	Unclear concerns
Basu et al [57]	Unclear	Unclear	Unclear	Unclear	Unclear concerns
Shankar et al [58]	Low	Low	Low	Unclear	Unclear concerns
Soltane et al [60]	Unclear	Low	Unclear	Unclear	Unclear concerns
Tagami et al [61]	Low	Low	Low	Unclear	Unclear concerns

### Data Synthesis Results

#### Data in the Included Literature

The number of participants across internal datasets varied significantly, with studies recruiting anywhere from 10 to 1005 patients diagnosed with lymphoma. Concurrently, the model development used a broad range of histopathological slides (count: 10-15,353). In most studies, the samples for model development were WSIs involving excised or biopsied tissues (38/41), with other samples using microscopic images (3/41). Most studies used HE-stained tissues (37/41), while others employed various immunohistochemical (IHC) staining methods (4/41). Some studies employed a multimodal analysis method that integrated pathological images with clinical information [25,35,50].

Among the included studies, most (29/41) used single-center data and few (10/41) used multicenter data. A small number of studies had unclear data sources, and the United States was the most common (12/41) source country.

#### Models in the Included Literature

The studies encompassed a variety of models, with the most prevalent being convolutional neural networks (CNNs), accounting for 30 out of 41 studies. A minority of studies employed support vector machines (SVMs) and random forests (2 studies each). The CNN architectures that were explored included Mobile Net, VGG-16, Hover-Net, U-Net, and ResNet-50. These newer CNNs generally incorporated multiple standardized blocks that featured layers for convolution, normalization, activation, and pooling [23]. One study stood out by leveraging transfer learning and integrating 17 distinct DL models to create a highly accurate platform, achieving a diagnostic accuracy rate of 100% [21]. This approach significantly bolstered the model’s ability to generalize. Another innovative study developed a novel architecture by applying a topological optimization method to the conventional VGG-16 model [26]. Some studies opted for a hybrid approach, combining traditional ML techniques with DL. They used CNNs for feature extraction, followed by decision tree–based methods for quantification and classification [24,26]. Notably, 1 study implemented a CNN framework grounded in Multiple Instance Learning (MIL), which autonomously concentrated on image patches from regions of interest within tumors, showcasing an advanced method for image analysis [32].

In the analysis encompassing various models, most studies predominantly used patches (33/41), with a subset operating at the WSI level (6/41). Two distinct aggregation approaches were implemented: premodeling and postmodeling aggregation. The premodeling method necessitated the creation of slide-level features prior to modeling, whereas the postmodeling approach entailed consolidating patch-level model outputs to formulate slide-level predictions. For models that used patch images as the basis for final modeling, it was essential to segment the original images into individual patches before proceeding with modeling. The patch sizes varied from 40×40 to 2048×2048 pixels, with the most frequently employed dimensions being 224×224 pixels (9/41) and 256×256 pixels (5/41). Subsequently, a variety of feature extraction techniques were applied, encompassing both handcrafted or predefined features (27/41) and features that were automatically learned by the models (12/41).

The handcrafted features encompassed a diverse spectrum of attributes, including texture, color, cellular, and nuclear morphological characteristics. These meticulously crafted features were predominantly employed as inputs for traditional ML algorithms, such as SVM and random forest models.

In contrast, learned features were predominantly extracted through the application of CNNs, which also frequently served as the classifier of choice. Ultimately, the outputs from the patch-level models were synthesized to develop predictive models. This aggregation was achieved through various methods, such as attention-based weighted averaging, concatenation, and more sophisticated embedding techniques. These included Fisher vector encoding and k-means clustering, with the process often culminating in the selection of the maximum value to enhance the predictive accuracy of the models [36,62].

Among the papers that specified magnification levels, the most prevalent were 20× (10/41) and 40× (17/41). A handful of studies employed varying magnifications strategically to pinpoint informative tissue regions and to enhance their modeling accuracy [16,17,40].

A limited number of models integrated histopathological data with other data modalities [25,35,50]. The multimodal approaches observed in the literature included the premodeling integration of unimodal features extracted independently, as well as the amalgamation of unimodal predictions from distinct models [63]. Additionally, transformer-based methods were frequently employed for encoding the intricate relationships between different modalities [64,65]. While attention-based methods have been used in the study of other malignancies for several years [66], their application in lymphoma research is a relatively recent development. Among the studies reviewed, 1 study stood out by using a variant of the transformer architecture to encode the interplay between medical imaging data and clinical records. This study introduced a novel personalized attention mechanism (PersAM) for the classification of lymphoma subtypes, marking a significant advancement in the field [25].

#### Analysis in the Included Literature

Most studies relied on internal validation (30/41), while external validation using independent lymphoma datasets was seldom conducted (11/41). In terms of internal validation, partial validation was typically executed through a 5-10–fold cross-validation approach. Some papers detailed the hyperparameter selection process using the training dataset, yet only reported evaluations on a test set derived from the same data source [24,33,67]. For external validation, models were trained on WSIs and subsequently validated on either WSIs (10/11) or tissue microarrays (TMAs) (1/11) from separate independent sources. Notably, the model of 1 study was externally validated against data from normal lymph node tissues [14]. In a particular instance, a model that achieved perfect validation accuracy (area under the receiver operating characteristic curve [AUC]=1.0) with internal validation underperformed on external cases, with an AUC ranging from 0.63 to 0.69. This discrepancy may stem from the sensitivity of ML algorithms to preprocessing steps, and neural networks, in accordance with statistical principles, necessitate a representative sample to ensure reliable inductive reasoning [12]. In another study, a comprehensive evaluation was conducted using polynomial, SVM, random forest, and decision tree classifiers to assess the efficacy of the proposed method [30].

Most models were assessed using accuracy or AUC, as well as other metrics, including sensitivity, specificity, hazard ratio, and the C-index. Even when studies reported sensitivity and specificity, CIs were not reported. In the internal validation results of all models, the AUC ranged from 0.75 to 0.99 and accuracy ranged from 68.3% to 100%. In models with external validation results, the AUC ranged from 0.93 to 0.99.

The burgeoning demand for AI methods in health care is undeniable, yet the lack of interpretability remains a significant impediment to their clinical adoption [68,69]. Enhancing the interpretability of AI models is crucial for fostering trust among medical professionals in the future AI systems they will rely upon. In a thorough analysis of the studies included, it was observed that the majority of studies (20/41) undertook efforts to analyze the interpretability of their models, with a notable minority (8/20) delving into visual interpretability analysis of the histopathological images that significantly influenced the model’s prognostic assessments. Several studies meticulously characterized the spatial distribution and interrelationships of typical cells, their nuclei, and the microenvironments within the regions of interest [24,32,36,42], thereby showcasing the interpretability of their AI systems. One study presented graphical features that were correlated with clinical prognostic information [38]. A handful of studies opted for traditional ML models, such as decision trees [26], which are inherently more transparent and align closely with human reasoning processes, thus facilitating a more intuitive understanding of the decision-making process.

#### Clinical Utility

Among the 41 models included, 17 were diagnostic models, 10 were prognostic models, 2 were models to detect gene translocation, and 12 were other prediction- and diagnosis-related information models. The tasks of these models included identification (8/41), classification (24/41), and segmentation (9/41).

In the field of AI-based diagnostic models for lymphoma histopathology, the most common subtypes included diffuse large B-cell lymphoma (DLBCL) (5/41), follicular lymphoma (FL) (8/41), chronic lymphocytic leukemia (CLL) (5/41), and mantle cell lymphoma (MCL) (8/41). Additionally, a small number of studies developed diagnostic models for Burkitt lymphoma, central nervous system lymphoma, high-grade lymphoma, HL, and T-cell lymphoma. A study using CNNs for cell segmentation of WSIs of NHL achieved an average diagnostic accuracy of 100%, 99.73%, and 99.20% for CLL, FL, and MCL, respectively [20]. Researchers have successfully harnessed the power of both DL and traditional ML to develop a diagnostic tool with remarkable accuracy rates ranging from 95% to 100% for identifying MCL, FL, and CLL. This cutting-edge approach involves the precise segmentation of cell nuclei and the meticulous measurement of key morphological features such as area, perimeter, eccentricity, and diameter [30].

In the realm of AI-based prognostic models for lymphoma histopathology, the most common subtypes include DLBCL (3/4), CLL (2/42), HL (2/41), and FL (2/41). Sereda et al [19] used DL-based cell detection on digital slides from patients with nodular lymphocyte-predominant Hodgkin lymphoma (NLPHL) to quantitatively assess the histological patterns of lymphocyte-predominant cells. They identified 6 key features of lymphocyte-predominant cell spatial patterns and achieved a high average precision in cell detection (mean 95.24%, SD 0.17%). Furthermore, they found a strong correlation between treatment response and the density and number of lymphocyte-predominant cells (*P*<.05). Several studies have identified independent prognostic factors for DLBCL and MCL by segmenting cell nuclei and calculating the geometric characteristics of each segmented nucleus [35,36]. Several studies [41-43] addressed the clinical challenge of large cell transformation in indolent B-cell lymphomas, such as FL and CLL. They trained a CNN to predict large cell transformation based on tumor cell morphology, including the small cell proportion, chromatin pattern, presence of distinct nucleoli, and proliferation index. The machine-generated quantifications demonstrated superior reproducibility compared to estimates made by pathologists and showed a stronger correlation with the outcome data. The precise assessment and evaluation of PD-L1 biomarkers are crucial for the targeted immunotherapy triage of cancer patients. Notably, Yan et al [48] developed an AI-based image analysis method that encompasses the detection, segmentation, and classification of PD-L1+ cells for the evaluation of PD-L1 expression in patients with DLBCL. This method has produced highly correlated quantitative results compared to the subjective assessments of pathologists. However, none of the prognostic models included T-cell lymphoma subtypes in the studies.

Regarding AI-based histopathological models for detecting gene translocations in lymphoma, 2 studies focused on DLBCL as the tumor type [39,56]. Their results showed that it is possible to predict MYC translocation based on morphology alone. This would allow simple and fast prescreening, saving about 34% of genetic testing using the current algorithm.

Overall, methodologically, all studies exhibited a high or unclear risk of bias, primarily due to limited analysis and incomplete reporting on participant recruitment. Most high-risk models (10/41) predominantly assigned high-risk classifications to participants. Almost all studies presented an unclear risk of bias in at least one domain, with the most frequent being participant selection (16/41) and statistical analysis (37/41). The primary reasons for this were insufficient analysis of participant recruitment and a lack of interpretability in outcome analyses. In the diagnostic models, the most frequently studied lymphoma subtypes were DLBCL, FL, CLL, and MCL, while in the prognostic models, the most common subtypes were DLBCL, FL, CLL, and HL. None of the prognostic models included T-cell lymphoma subtypes in the studies. In the internal validation results of all models, the AUC ranged from 0.75 to 0.99 and accuracy ranged from 68.3% to 100%. In models with external validation results, the AUC ranged from 0.93 to 0.99.

### Sensitivity Analysis

To further evaluate the sensitivity of our conclusions, we conducted a sensitivity analysis by selecting diagnostic models from the literature we included and performing a QUADAS-AI diagnostic evaluation. The results are presented in Multimedia Appendix 4.

Our findings revealed that out of the 17 diagnostic models considered, 15 were rated as high-risk models and 2 were deemed unclear. Most high-risk models were classified as such primarily due to their use of nonpublic datasets (13/17). A minority of studies (2/17) were rated as high risk because they failed to provide a clear description of their data sources. Therefore, we used QUADAS-AI to evaluate the diagnostic models and concluded that all the methodologies included in the diagnostic models were biased.

To further assess the sensitivity of our conclusions, we considered that the distribution of lymphoma subtypes varies by region, which could potentially bias the results. Therefore, we only included studies from the United States, as it was the most common source country (12/41). We found that in the United States, the most common subtypes in AI-based diagnostic models for lymphoma histopathology were DLBCL (5/12), FL (2/12), and MCL (2/12). Regarding AI-based prognostic models for lymphoma histopathology, the most common subtypes were DLBCL (2/12), CLL (3/12), and FL (1/12). In the models for detecting gene translocations in lymphoma histopathology, 1 study focused on DLBCL. These findings are largely consistent with our previous conclusions.

## Discussion

### Current Status of AI in Lymphoma Assessment

AI has significantly enhanced the precision of lymphoma diagnostics by eliminating the subjectivity often associated with human observation [16,26]. For instance, a study by Achi et al [13] demonstrated the power of CNN in accurately distinguishing between various lymphoma subtypes. Their diagnostic model, designed for 4 distinct lymphoma categories (benign lymph nodes, DLBCL, Burkitt lymphoma, and small lymphocytic lymphoma), achieved an impressive 95% accuracy rate in image prediction. Notably, in a multicenter study [21], the diagnostic accuracy of AI for DLBCL reached 100%.

Moreover, AI provides invaluable insights into the tumor microenvironment, enabling the identification and quantification of image features that surpass simple density assessments. It delves into higher-order relationships and offers a quantitative evaluation of lymphocyte aggregation patterns and the complex interplay between tumor regions. Such capabilities are pivotal for advancing cancer clinical research and the development of new therapeutics [36,70].

However, methodologically, the risk assessments of the included studies in this review were all rated as high or unclear, primarily due to incomplete reporting, absence of detailed patient source information, and inadequate explanation of the predictors used. This highlights the need to expedite the clinical translation of AI in lymphoma diagnosis, ensuring that these advanced tools are rigorously validated and seamlessly integrated into clinical practice.

Frequently omitted details include the precise origin of patient data, the total number of patients involved, the quantity of samples or images used, and the techniques employed for tissue processing and digitization. Most studies reported data from single centers, and this scarcity may stem from AI researchers not dedicating sufficient effort to comprehend these images, whether for training purposes or external validation. Information about the predictors (histopathology images and their features) was generally better reported; however, there remains an absence or inadequacy of the detailing of certain critical aspects. For example, it is often unclear whether the investigators assessed the predictors without knowing the outcomes or whether all histopathology images were processed uniformly, which could have introduced bias. Moreover, some researchers rely on a limited dataset and analyze a single test data split without implementing methods to mitigate overfitting and model optimism, such as cross-validation or external validation. These limitations are prevalent in lymphoma AI research, resulting in weak validation and an elevated risk of bias within the models.

Code sharing is crucial for enhancing the reproducibility of research findings and mitigating the effects of incomplete reporting. However, in the review of 41 papers, only 9 included codes, and the data in the other studies were either incomplete or difficult to access. To foster better reproducibility, code repositories should provide comprehensive documentation. This should include instructions for setting up the environment, an overview of the code’s functionality, guidance on how to produce results, and, of course, the code itself [14,17,21,26,38,44,46,48,58].

Several studies are dedicated to enhancing the interpretability of DL tools by employing existing methods. These include post hoc techniques and supervised ML models that interpret the outcomes after DL models have generated predictions [71,72]. In the realm of AI research focused on lymphoma, most current studies offer personalized interpretability for analysis. This includes visual attention heatmaps and traditional ML highlighting the spatial locations of key feature areas and their interrelationships. However, traditional ML, often crafted in partnership with domain experts, can provide greater interpretability as it relies on manually engineered features. Despite this advantage, the process of handcrafting features is inherently challenging and complex. It demands a substantial time commitment from pathologists or oncologists, who are responsible for developing these methods.

In recent years, there has been a notable increase in hybrid approaches that combine DL with handcrafted strategies. These methods might involve using DL algorithms for the preliminary detection of cells or elements, followed by the application of easily interpretable traditional ML techniques for making predictions. By doing so, they harness domain knowledge to ensure the biological interpretability of the approach [73].

### Development of the Field

The domain of AI in lymphoma histopathology diagnosis and prognosis is experiencing rapid growth, with a notable surge in scholarly publications since 2019. Most of these studies have leveraged deep neural networks for automated feature extraction and classification. In contrast, a smaller subset of research has employed conventional ML algorithms [24,30,33,40]. Recent investigations have expanded their scope to encompass a wider array of diagnostic outcomes, such as identifying specific lymphoma subtypes [17,21], predicting prognosis [35,36], and detecting genetic translocations [39,56].

Despite advancements regarding the role of AI in lymphoma research, there has not been a noticeable trend toward larger datasets, either in the number of slides analyzed or the number of participants involved. While there is no indication that more recent studies have adopted stricter internal validation methods, there is a positive shift toward increased external validation. Prior to 2019, no studies incorporated external validation on lymphoma data; however, 10 recent publications have done this. Although these validations often involve limited data, their inclusion signifies a step forward in research methodology.

These external validations are essential for the practical application of AI models in clinical settings, as they must be robust enough to handle the visual diversity present in data from various sources. This diversity can fluctuate both between and within different data centers over time. As the field evolves, we expect to see an increase in studies that rigorously validate their models against larger, high-quality, independent datasets. This includes transparent reporting on patient recruitment and selection processes, histopathology slide preparation, and digitization techniques. Such practices will be instrumental in mitigating the biases, limited reproducibility, and restricted generalizability that currently plague much of the research in this domain.

In the realm of oncology, there has been a marked surge in the number of published multimodal research studies since 2019. Notably, this growth includes only a handful of studies focused on multimodal research in lymphoma. Histopathological examination of tissue sections continues to be the cornerstone for cancer diagnosis, yet even seasoned pathologists often seek support from biomarker assays to enhance diagnostic accuracy. Multimodal research, which amalgamates diverse data types, such as genomics, proteomics, transcriptomics, and clinical data, has been pivotal in steering the trajectory of cancer research. This approach not only consolidates information from various sources but also transforms it, offering novel insights [74,75]. For instance, a multimodal framework can facilitate a dual-modality analysis where a pathological image is processed to yield outputs from another domain, such as genetic sequencing or different imaging formats [63]. Such a model, when adeptly trained, can be leveraged to analyze pathological images from patients without overt medical conditions, extracting valuable indicators pertinent to precision medicine, including genetic sequences. As we anticipate advancements in high-throughput technologies, alongside the fields of transcriptomics, metabolomics, and proteomics, the future holds promise for an increase in the integration of multidimensional omics data with histopathological images in multimodal analyses.

This integration is poised to significantly bolster the clinical utility of AI, enabling more precise and personalized treatment strategies in oncology. By harnessing the power of multimodal research, we can expect a future where AI plays an even more integral role in clinical decision-making, thereby enhancing patient outcomes.

### Current Limitations and Future Recommendations

A considerable amount of published research lacks the necessary clinical and pathological details to evaluate potential biases effectively. As a result, it is imperative for AI researchers to meticulously document the origins of their data. This transparency is crucial for understanding the variability within the dataset and for determining whether this diversity has been adequately addressed in the research methodology. Additionally, the modeling and analytical techniques employed must be thoroughly described to ensure the reliability and reproducibility of the findings.

To further improve reproducibility, we recommend that researchers provide code and data whenever possible. Digital pathology studies on lymphoma are currently constrained by the lack of publicly available data. Furthermore, WSIs from different centers can lead to significant heterogeneity in image data due to differences in scanning equipment across various centers. This variability can introduce confounding factors that complicate the task of developing robust AI models and assessing their generalizability. Such limitations can increase the risk of bias and confusion in research findings.

To mitigate these issues, during the image preprocessing stage, it is essential to diligently address and eliminate confounding factors arising from variations in staining, the presence of bubbles, and other artifacts. This meticulous attention to detail will enhance the accuracy and reliability of AI-driven diagnostic and analytical tools.

For AI to be clinically valuable, rigorous validation is paramount, particularly considering the constraints inherent in existing datasets. We recommend that researchers employ comprehensive analytical methods, such as cross-validation and external validation, to substantiate the robustness of their findings and the capacity of their models to extend to new datasets. Moreover, it is essential to report CIs for results, with a focus on the 95% CI, especially when comparing various models. This practice aids in discerning whether observed differences in model performance are genuinely significant or merely a product of random variation. By doing so, researchers can make more informed decisions about the efficacy and reliability of different AI models in clinical settings.

Researchers are recommended to follow regulatory guidance and standardized processes in the field of medical AI, such as reporting guidelines and quality assessment tools, such as QUADAS-AI, which provide a specific framework for assessing the risk of bias and the applicability of studies of diagnostic test accuracy in AI centers.

Moreover, a lack of interpretability is a barrier to the clinical adoption of AI. Therefore, we recommend that researchers strive to demonstrate the interpretability of their models to enhance the understanding and trust of clinical and pathological professionals.

Lymphoma is a diverse type of blood cancer that includes a variety of subtypes. From the literature we have reviewed, the current application of AI in lymphoma histopathology has primarily been focused on the diagnosis and prognosis of B-cell lymphoma. There are relatively fewer AI models for HL or T-cell lymphoma and other rare subtypes. This could be attributed to the higher incidence of B-cell lymphomas compared to other subtypes [1,2]. However, given the high aggressiveness and heterogeneity of T-cell lymphoma and HL [76], we hope that in future model development, more researchers will develop AI models for T-cell lymphoma and HL from a multidimensional clinical perspective.

### Conclusion

Methodologically, the diagnostic and prognostic models of AI applied to lymphoma histopathology were evaluated, and the models were found to be biased. The enhancement of the accuracy of AI models and the acceleration of their clinical translation hinge on several critical aspects. These include the comprehensive reporting of data sources, the diversity of datasets, the study design, the transparency and interpretability of AI models, the use of cross-validation and external validation, and adherence to regulatory guidance and standardized processes in the field of medical AI.

## Appendix

Multimedia Appendix 1 Literature search strategies in PubMed, Cochrane Library, and Web of Science.

Multimedia Appendix 2 Description of quality assessment based on Quality Assessment of Diagnostic Accuracy Studies-AI (QUADAS-AI) domains used to evaluate the methodological quality of the studies included.

Multimedia Appendix 3 Prediction Model Risk of Bias Assessment Tool (PROBAST) table.

Multimedia Appendix 4 Quality Assessment of Diagnostic Accuracy Studies-AI (QUADAS-AI) table.

Multimedia Appendix 5 PRISMA (Preferred Reporting Items for Systematic Reviews and Meta-Analyses) 2020 checklist.

## Abbreviations

AI: artificial intelligence
AUC: area under the receiver operating characteristic curve
CLL: chronic lymphocytic leukemia
CNN: convolutional neural network
DL: deep learning
DLBCL: diffuse large B-cell lymphoma
FL: follicular lymphoma
HE: hematoxylin-eosin
HL: Hodgkin lymphoma
IHC: immunohistochemical
MCL: mantle cell lymphoma
ML: machine learning
NHL: non-Hodgkin lymphoma
PROBAST: Prediction Model Risk of Bias Assessment Tool
QUADAS-AI: Quality Assessment of Diagnostic Accuracy Studies-AI
SVM: support vector machine
WSI: whole slide image
