# Supplementary material for: Artificial Intelligence in Lymphoma Histopathology: Systematic Review
Source: J Med Internet Res. 2025 Feb 14;27:e62851. doi: 10.2196/62851 (PMC11888075; doi:10.2196/62851)
Supplement: Multimedia Appendix 1 [file jmir_v27i1e62851_app1.docx]

**Multimedia Appendix 1.** Literature search strategies in PubMed, Cochrane Library, and Web of Science.

**PubMed**

| Search | **PubMed Query –August 30, 2024** | Items found |
| --- | --- | --- |
| #6 | #1 AND #5 | 2762 |
| #5 | #2 OR #3 OR #4 | 896607 |
| #4 | "Neural networks, computer"[Mesh Terms] OR "computer neural network"[Title/Abstract] OR "computer neural networks"[Title/Abstract] OR “models” neural network"[Title/Abstract] OR "model neural network"[Title/Abstract] OR "connectionist models"[Title/Abstract] OR "connectionist model"[Title/Abstract] OR "Perceptions"[Title/Abstract] OR "computational neural networks"[Title/Abstract] OR "computational neural network"[Title/Abstract] | 78435 |
| #3 | **"Machine learning"[** Mesh Terms**] OR "deep learning"[Title/Abstract] OR "DL"[Title/Abstract] OR "ML"[Title/Abstract] OR "supervised machine learning"[Title/Abstract] OR "support vector machine"[Title/Abstract] OR "unsupervised machine learning"[Title/Abstract]** | 718997 |
| #2 | "artificial intelligence"[MeSH Terms] OR "intelligence artificial"[Title/Abstract] OR "computer reasoning"[Title/Abstract] OR "machine intelligence"[Title/Abstract] OR "intelligence machine"[Title/Abstract] OR "computational intelligence"[Title/Abstract] OR "computer vision systems"[Title/Abstract] OR "computer vision system"[Title/Abstract] OR "system computer vision"[Title/Abstract] | 245279 |
| #1 | (((((lymphoma [MeSH Terms]) OR (Lymphomas[Title/Abstract])) OR (Lymphoma, Malignant[Title/Abstract])) OR (Lymphomas, Malignant[Title/Abstract]))OR(Malignant Lymphoma[Title/Abstract]))OR(Malignant Lymphomas[Title/Abstract]) | 208785 |

**Cochrane Library**

| Search | **Cochrane Query –August 30, 2024** | Items found |
| --- | --- | --- |
| #14 | #10 AND #13 | 12 |
| #13 | #11OR#12 | 5706 |
| #12 | (Lymphomas): ti, ab,kw OR (Malignant Lymphomas):ti,ab,kw OR (Lymphomas, Malignant):ti,ab,kw OR (Reticulolymphosarcomas):ti,ab,kw OR (Reticulolymphosarcoma):ti,ab,kw | 1627 |
| #11 | **MeSH descriptor:** [**Lymphoma**] **Explode all trees** | 4653 |
| #10 | **#3OR#6OR#9** | 9432 |
| #9 | **#7OR#8** | 1603 |
| #8 | **(Connectionist Model): ti, ab,kw OR (Computer Neural Networks):ti,ab,kw OR (Neural Network Model):ti,ab,kw OR (Connectionist Models):ti,ab,kw OR (Neural Network, Computer):ti,ab,kw OR (Computational; Perceptions):ti,ab,kw OR (Network, Computer Neural):ti,ab,kw OR (Models, Neural Network; Network Model, Neural):ti,ab,kw** | 2295 |
| #7 | **MeSH descriptor: [Neural Networks, Computer] explode all trees** | 641 |
| #6 | **#4OR#5** | 5053 |
| #5 | **(Learning, Machine): ti, ab,kw OR (Transfer Learning):ti,ab,kw OR (Learning, Transfer):ti,ab,kw** | 4741 |
| #4 | **MeSH descriptor: Machine Learning Explode all trees** | 1009 |
| #3 | #1OR#2 | 5094 |
| #2 | (Acquisition (Computer)): ti, ab,kw OR (Acquisition, Knowledge (Computer)):ti,ab,kw OR (Vision System, Computer):ti,ab,kw OR (Intelligence, Machine):ti,ab,kw OR (Computational Intelligence):ti,ab,kw OR ((Computer Reasoning):ti,ab,kw OR (Knowledge Representation (Computer)):ti,ab,kw OR (Knowledge Representations (Computer)):ti,ab,kw | 251 |
| #1 | MeSH descriptor: [Artificial Intelligence] explode all trees | 3297 |

**Web of Science**

| Search | **Web of science Query –August 30, 2024** | Items found |
| --- | --- | --- |
| #3 | #1 AND #2 | 1183 |
| #2 | ((((((((TS= (Artificial intelligence)) OR TS= (machine learning))) OR TS=(deep learning))) OR TS=(neural network)) OR TS=(AI )) OR TS=(Supervised Machine Learning)) OR TS=(Support Vector Machine)) OR TS=(Unsupervised Machine Learning)) OR TS=(Computer Neural Network) | 1397935 |
| #1 | ((((TS=(lymphoma)) OR TS=(Lymphomas)) OR TS= (Lymphoma, Malignant)) OR TS=(Lymphomas, Malignant)) OR TS=(Malignant Lymphoma) | 246122 |
