# Supplementary material for: Artificial Intelligence in Lymphoma Histopathology: Systematic Review
Source: J Med Internet Res. 2025 Feb 14;27:e62851. doi: 10.2196/62851 (PMC11888075; doi:10.2196/62851)
Supplement: Multimedia Appendix 2 [file jmir_v27i1e62851_app2.docx]

**Multimedia Appendix 2.** Description of quality assessment based on Quality Assessment of Diagnostic Accuracy Studies-AI (QUADAS-AI) domains used to evaluate the methodological quality of the studies included.

| **Domain** | **Subject selection** | **Index test (AI)** | **Reference standard** | **Workflow** |
| --- | --- | --- | --- | --- |
| **Concern** | **Signalling question:**   - **Accurately characterize the source, size and quality of input data alongside clear patient eligibility criteria？** - **Was it derived from open-source datasets?** - **Present the rationale and breakdown of its training, validation, and test sets?** - **Whether to perform image pre-processing?**   **Provide the scanner model information used to acquire imaging data?** | **Signalling question:**   - **Was external verification performed?** | **Signalling question:**   - **Was the reference standard likely to correctly classify the target condition?** | **Signalling question:**   - **Was the time between the index test and the reference standard reasonable?** |
| **Concerns regarding “risk of bias”** | **Risk of bias is judged as “low”, “high”, or “unclear”.**   - **If all signalling questions for a domain are answered “yes” then risk of bias can be judged “low”.** - **If any signalling question is answered “no” this flags the potential for bias. Review authors then need to have in-depth discussions to judge risk of bias.**   **The “unclear” category should be used only when insufficient data are reported to permit a judgment.** | | | |
